# Central Retinal Artery Occlusion After Intravitreal Injection of Faricimab: A Case Report

**DOI:** 10.7759/cureus.91170

**Published:** 2025-08-28

**Authors:** Hiromasa Hirai, Shinnosuke Yasuda, Sho Imoto, Hiroki Tsujinaka, Satoru Kase

**Affiliations:** 1 Ophthalmology, Nara Medical University, Kashihara, JPN

**Keywords:** angiopoietin-2, anti-vascular endothelial growth factor drug, central retinal artery occlusion, central retinal vein occlusion, faricimab, vascular endothelial growth factor

## Abstract

Faricimab is a relatively novel agent as a bispecific antibody that inhibits both vascular endothelial growth factor-A and angiopoietin-2. Although it's effective for several retinal diseases, several rare adverse events, such as retinal vasculitis, have been reported. We present a case of central retinal artery occlusion (CRAO) following intravitreal injection of faricimab in a 74-year-old woman treated for central retinal vein occlusion. A sudden decrease in visual acuity occurred 24 hours after the second injection. Her best-corrected visual acuity was counting fingers at 30 cm. No anterior chamber inflammation was observed. Fundus examination showed retinal whitening at the posterior pole. Optical coherence tomography revealed intraretinal edema, and fluorescein angiography showed mild perfusion delay without leakages from retinal vessels. She was diagnosed with CRAO. After one month, her vision remained at 0.01. This case highlights that CRAO may occur as a rare but serious vascular adverse event following faricimab injection. Ophthalmologists should be aware of this potential complication and counsel patients accordingly.

## Introduction

Anti-vascular endothelial growth factor (VEGF) drugs are commonly used for the treatment of age-related macular degeneration, diabetic macular edema, and retinal vein occlusion [1]. Faricimab is a bispecific antibody that binds and neutralizes both VEGF-A and angiopoietin-2 (Ang-2), thereby exerting potent anti-angiogenic and vascular-stabilizing effects [2]. Ophthalmologists have conducted intravitreal injection of faricimab in local clinics as well. However, several recent reports and post-marketing surveillance have indicated that it may cause side effects, including retinal vasculitis [2-5].

The United States prescribing information for faricimab (updated by Genentech) notes an estimated incidence of occlusive retinal vasculitis of 0.06 per 10,000 injections [5]. Although this figure is based on United States data, it highlights the potential global relevance of such adverse events, including in Japan. On the other hand, retinal artery occlusion is a serious ophthalmic emergency disorder that can lead to permanent vision loss [6]. There is still no established treatment, and delayed medical attention often results in poor visual prognosis. Although there are a few reports of retinal artery occlusion following anti-VEGF drug administration [7-9], there have been no individual case reports of arterial occlusion following faricimab administration.

Here, we report a case of central retinal artery occlusion (CRAO) that developed after intravitreal injection of faricimab for central retinal vein occlusion (CRVO).

## Case presentation

A 74-year-old woman presented to her local eye clinic with decreased vision in her left eye. She had a history of unilateral kidney donor nephrectomy, mild dyslipidemia, and mild depression. She had no ophthalmic history. She had been prescribed mirtazapine by her local doctor for mild depression. Her decimal best corrected visual acuity in the left eye was 0.9, and macular edema due to CRVO was observed. She received one intravitreal injection of faricimab and panretinal photocoagulation. After the treatment, the macular edema improved.

Seven months later, macular edema recurred. Thus, a second intravitreal injection of faricimab was administered. The injection was completed without issues, and the follow-up visit the next morning showed no significant changes. However, she noticed a sudden decrease in vision in her left eye in the evening. Since her left vision did not improve, she revisited the local eye clinic two days later. Betamethasone sodium phosphate 0.1% eye drops were given four times daily to her left eye. Additionally, brinzolamide-timolol maleate eye drops (brinzolamide 1% and timolol maleate 0.5%) were prescribed for the left eye, to be administered twice daily. She was referred to our ophthalmology department the following day. The timeline summarizing the sequence of events is shown in Table 1. 

**Table 1:** Timeline of clinical course before presentation at our ophthalmology department CRVO, central retinal vein occlusion

Timeline	Event
First presentation at local clinic	CRVO and macular edema diagnosed at the local eye clinic
	First intravitreal injection of faricimab + Panretinal photocoagulation
	The macular edema improved after treatment
Second presentation at local clinic (7 months from CRVO onset); Day 0	Recurrence of macular edema observed
	Second intravitreal injection of faricimab
Day 1 morning	Showed no significant changes
Day 1 evening (24 hours after injection)	Sudden decrease in vision
Day 3	Revisited the local eye clinic
Day 4	Referred to our ophthalmology department

**Figure 1:**
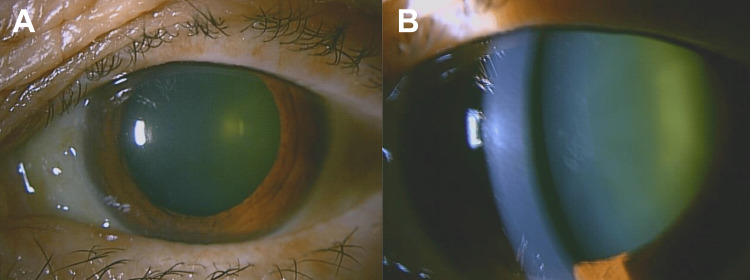
Image of anterior segment of the left eye at the initial visit to our department (A) No ciliary injection is observed. (B) No corneal keratic precipitates or anterior chamber inflammation are observed.

At her initial visit to our department, her best corrected visual acuity was 30cm counting fingers and 1.2 in her left and right eye, respectively. Her intraocular pressure (IOP) was 17 mmHg in the left eye and 19 mmHg in the right eye. Neither corneal keratic precipitates nor anterior chamber inflammation were observed (Figure 1).

**Figure 2:**
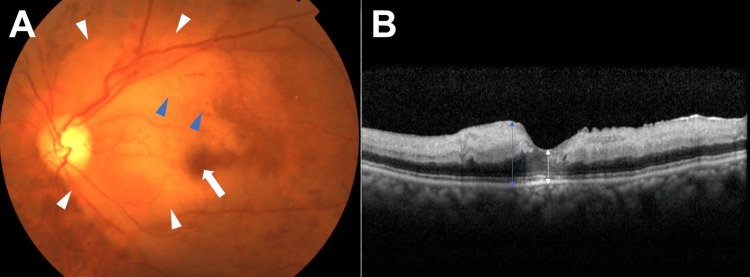
Fundus photograph of the left eye (A) and vertical scan OCT image of the left eye (B) at the initial visit to our department. (A) Whitening of the posterior pole retina is observed (white arrowheads). Cherry red spot is observed at the macula (white arrow). Retinal vessel with white sheathing is also observed (blue arrowheads). (B) Edema of the inner retinal layers is observed. Central retinal thickness is 252 μm (white arrow). Retinal thickness at the thickest point is 500 μm (blue arrow).

**Figure 3:**
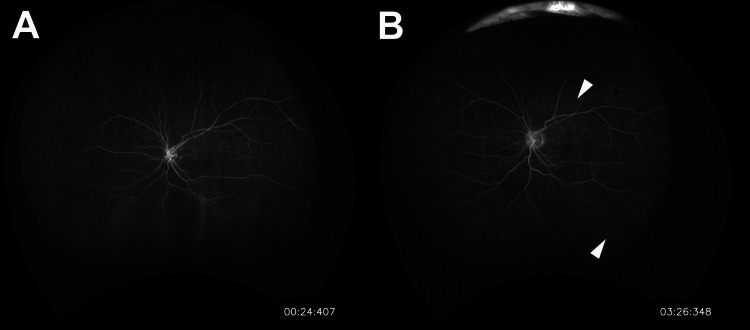
Wide-angle fundus photograph of fluorescein angiography at the initial visit to our department. (A) Wide-angle fundus photograph of the early arterial phase fluorescein angiography (24 seconds). The onset of perfusion is slightly delayed. (B) Wide-angle fundus photograph of the late phase fluorescein angiography (3 minutes and 26 seconds). No perfusion defects in the retinal arteries due to thrombosis or fluorescein leakage from the retinal vessels are observed. Photocoagulation scars are observed around the peripheral retina (white arrowheads).

Fundus examination in the left eye without vitreous haze showed whitening of the posterior pole retina consistent with cherry-red spot (Figure 2A). Optical coherence tomography (OCT) in the left eye showed inner retinal layer edema surrounding the macula (Figure 2B). Fluorescein angiography in the arterial phase showed mild perfusion delay without fluorescein leakages from retinal vessels (Figure 3). Blood tests showed a slight increase in total cholesterol (300 mg/dL).

The patient was diagnosed with CRAO. Since three days had passed from the onset and the reperfusion was achieved, the topical eye drops (betamethasone and brinzolamide-timolol maleate) were continued with no additional medication. Invasive interventions such as intravenous tissue plasminogen activator therapy or hyperbaric oxygen therapy were not pursued due to the delayed presentation and limited evidence of efficacy in such cases. There were no definite signs of inflammation, and thus systemic corticosteroid therapy was not indicated. 

**Figure 4:**
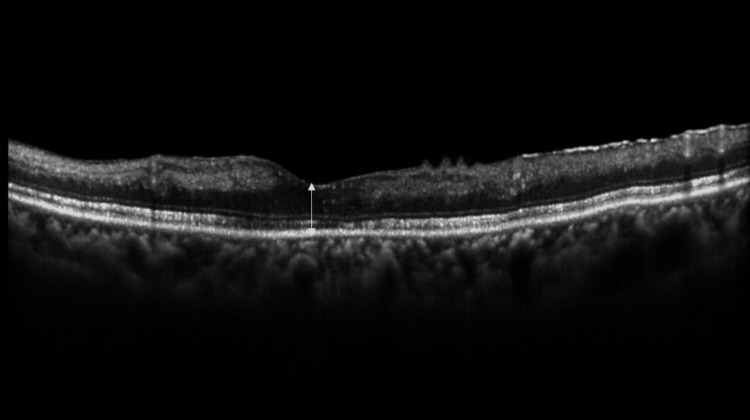
Vertical scan OCT image of the left eye one month after first presentation at our department. Edema in the inner retinal layer seen at the initial visit is resolving. Central retinal thickness is 195 μm (white arrow). OCT: optical coherence tomography

One month later, her left decimal best corrected visual acuity was 0.01. Although she had discontinued the eye drops one week ago on her own, the left IOP was 20 mmHg, and no anterior chamber inflammation was observed. The retinal inner layer had thinned (Figure 4). 

To evaluate potential thrombotic factors, additional systemic examinations, including carotid ultrasound and brain MRI, were planned. However, due to family circumstances, the patient declined further testing. This case has been reported to the manufacturer as a suspected adverse drug reaction. The causality between intravitreal faricimab and CRAO was considered “probable” according to clinical judgment.

## Discussion

In this case, CRAO occurred in the eye with CRVO about 24 hours after the second intravitreal injection of faricimab. There are a few reports of CRAO or BRAO occurring during CRVO treatment. Notably, ischemic CRVO has been reported to increase the risk of retinal artery occlusion [10]. However, in this case, the retinal perfusion was relatively well maintained. Although it is also known that patients with a history of hypertension or cardiovascular disease have an increased risk of retinal artery occlusion [6], the patient had no such direct risk factors other than mild dyslipidemia in this case.

Retinal occlusive vasculitis is a rare complication after anti-VEGF therapy. Recently, there have been several reports of uveitis or retinal occlusive vasculitis following intravitreal injection of faricimab [2-5]. However, CRAO was noted in this case, while no significant anterior segment inflammation was observed. A previous report described that occlusive vasculitis following various anti-VEGF agents developed with a mean onset time of 26.1 days and was suggested to be triggered by a type III or IV hypersensitivity reaction, leading to sterile intraocular inflammation [2]. In contrast, in the present case, CRAO occurred suddenly 24 hours after faricimab injection, and fluorescein angiography showed no clear signs of retinal vascular inflammation. These discrepancies suggest that the mechanism in this case may differ from delayed hypersensitivity-mediated vasculitis.

It is known that IOP transiently increases with the injection of intravitreal medication [11]. Persistent high IOP can potentially lead to arterial occlusion. However, no issues were observed at the end of the injection or the follow-up visit the next morning. Thus, the temporary IOP elevation was unlikely to be the cause in this case.

There are several reports of retinal artery occlusion following other anti-VEGF intravitreal injections [7,8,12-14]. Although the mechanism is unclear due to the small number of cases, a previous study has suggested that bevacizumab, an anti-VEGF agent, could lead to narrowing of retinal vessel diameters [12]. Interestingly, there have been several reports of retinal arterial occlusion without apparent retinal vasculitis following brolucizumab administration [15,16]. Kusuhara et al. also noted sites of arterial occlusion without angiographic signs of active vasculitis in eyes with brolucizumab-associated occlusive vasculitis [16]. The strong and sustained VEGF inhibition by therapeutic agents may induce endothelial dysfunction and thrombosis. In post-marketing surveillance of faricimab, a case of retinal artery occlusion has been reported [1], but no previous case of retinal artery occlusion following faricimab injection for CRVO has been published. Faricimab uniquely exerts dual inhibition of VEGF-A and Ang-2, resulting in sustained fluid removal effects in retinal vascular diseases [17]. These combined effects may have had a more pronounced impact on retinal arteries in our patient, potentially leading to the sudden arterial occlusion. With the increasing use of faricimab, awareness of this potential complication is important, and further accumulation of cases will be essential to clarify the underlying mechanisms.

## Conclusions

This was a rare case of CRAO occurring shortly after intravitreal injection of faricimab. Although the precise mechanism remains uncertain, possible contributors include embolic events, immunologic reactions, or vascular effects related to dual VEGF-A and Ang-2 inhibition. Ophthalmologists should remain cautious for acute vision loss after anti-VEGF therapy and educate patients that CRAO constitutes an ophthalmic emergency requiring immediate evaluation.
